# Supplementary material for: Effects of Feed Supplementation on Nesfatin-1, Insulin, Glucagon, Leptin, T3, Cortisol, and BCS in Milking Ewes Grazing on Semi-Natural Pastures
Source: Animals (Basel). 2021 Mar 4;11(3):682. doi: 10.3390/ani11030682 (PMC7999527; doi:10.3390/ani11030682)
Supplement: Supplementary file 1 [file animals-11-00682-s001.pdf]

Effects of Feed Supplementation on Nesfatin-1, Insulin, Glucagon, Leptin, T3, Cortisol, and BCS in Milking Ewes Grazing on Semi-Natural Pastures

Olimpia Barbato, Elena De Felice, Luca Todini, Laura Menchetti, Alessandro Malfatti, Paola Scocco

Material and methods

Experimental trial

The trial was carried out on 24 Comisana x Appenninica pluriparous lactating ewes. Animals at the moment of the offspring separation (near 40 days post-partum) were divided in two homogeneous groups as far as body weight (BW) and Body Condition Score (BCS), parity, days of lactation and milk yield. All animals were 3 years old, had 2 parity with 1 lamb/pregnancy. One group (supplemented group, SUP, n=12, mean BW 49.4±6.1, mean BCS 2.31±0.3; mean milk production 306±64 mL/d) was supplemented with 600 g/day/animal of corn and barley (1:1), while the other group (control unsupplemented group, UNS, n=12, mean BW 50.5±5.1, mean BCS 2.27±0.3; mean milk production 297±63 mL/d) fed only with the pasture.

Hormone levels and BCS were evaluated before the supplementation on 7 July (T0) and, after this date, every 9-10 days until 22 August (T1-T5). The date of 22 August corresponded to the pre-mating period, when males were introduced in the flock and all females were dried. Experimental trial scheme is showed in Figure S1

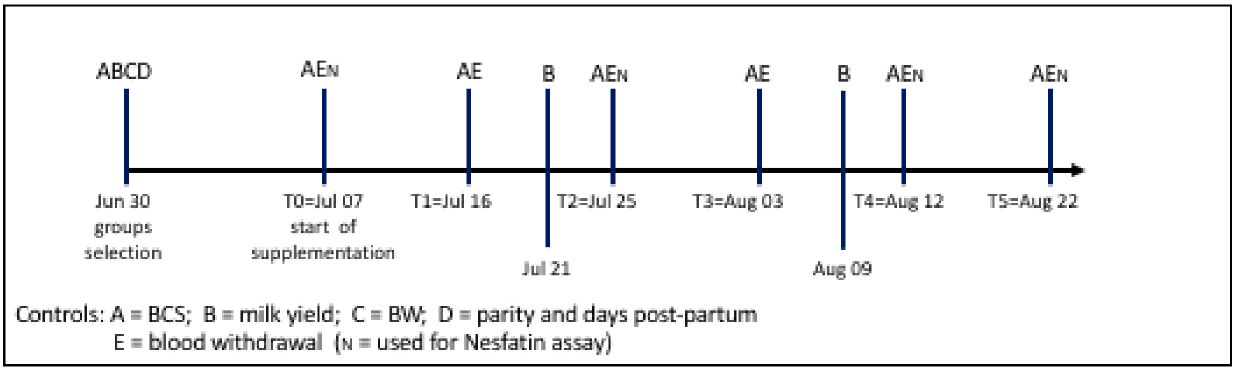

Figure S1. Experimental trial scheme

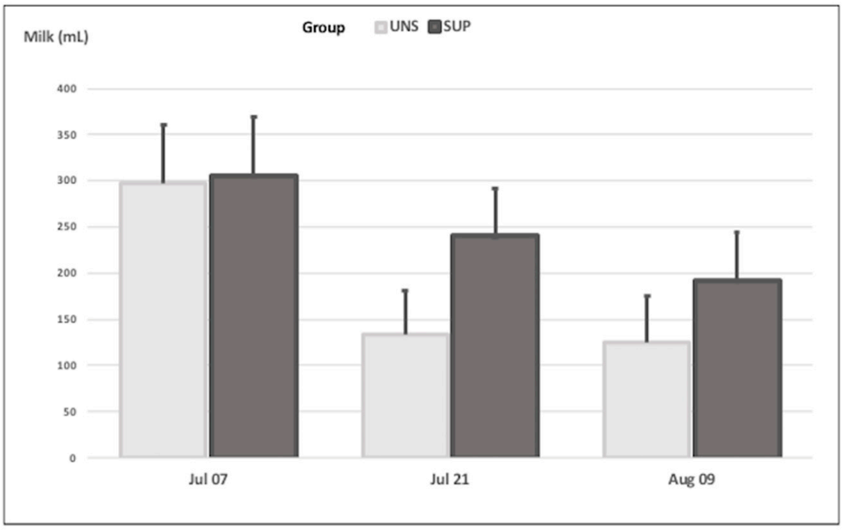

Figure S2. Means and standard errors of milk production at morning milking (mL) in control (UNS) and supplemented (SUP) lactating sheep. Milk yield was recorded as manually collected milk by each animal.

Table S1. Mean and standard error (SE), and median (Mdn) with interquartile range (IQR) of Nesfatin-1 concentrations in control (UNS) and supplemented ewe (SUP) ewe before the administration of supplementation (T0), and after 20 (T2), 40 (T4), and 50 (T5) days of supplementation.

GROUP	*TIME*							
	T0		*T2*		*T4*		*T5*	
	Mean ±SE	*Mdn (IQR)*	*Mean ±SE*	*Mdn (IQR)*	*Mean ±SE*	*Mdn (IQR)*	*Mean ±SE*	*Mdn (IQR)*
UNS	104.43±66.53	16.57 (12.96, 61.76)	194.09±101.34	43.93 (14.70, 123.54)	200.81±95.25	36.54 (14.96, 263.21)	139.22±104.90	47.71 (11.89, 358.07)
SUP	93.86±75.65	12.23 (10.46, 32.75)	64.64±49.49	15.32 (13.34, 17.85)	94.96±74.82	14.90 (12.41, 37.64)	50.21±37.96	12.90 (10.61, 70.71)
